# The Beneficial Effects of Ethanol Extract of the Microalga *Phaeodactylum tricornutum* in Alcoholic Liver Disease

**DOI:** 10.3390/ijms26083851

**Published:** 2025-04-18

**Authors:** Dae Yoon Kim, Seung Cheol Shin, Gab Jung Kim, Jae-In Eom, Cheol-Ho Han, Cheol-Ho Pan, Jae Kwon Lee

**Affiliations:** 1 College of Pharmacy, Chungbuk National University, Cheongju 28160, Republic of Korea; lnbsky@naver.com; 2 Department of Biology Education, College of Education, Chungbuk National University, Cheongju 28160, Republic of Korea; ssfe8435@chungbuk.ac.kr (S.C.S.); kgj950804@gmail.com (G.J.K.); 3 Microalgae Ask Us Co., Ltd., Gangneung 25441, Republic of Korea; umjaein@maus2020.com (J.-I.E.); hancheolho@maus2020.com (C.-H.H.)

**Keywords:** alcoholic liver disease, *Phaeodactylum tricornutum*, microalgae, cytokines

## Abstract

Alcoholic liver disease (ALD) is a condition resulting from liver damage linked to excessive drinking over a brief duration. It poses a significant public health challenge globally, with its prevalence and morbidity rising annually due to escalating rates of alcohol abuse, which adversely affect human health. *Phaeodactylum tricornutum* (PT), a diatom species of microalgae, is reported to possess active components that provide anti-inflammatory and antioxidant benefits. This study aimed to investigate the preventive and therapeutic effects of PT extract on ALD. To address our purpose, we used ethanol diet induced live disease model. Mice fed an ethanol diet showed less weight gain and higher levels of AST and ALT compared to those fed a regular diet. PT extract suppressed the inhibition of weight gain and the increase in AST/ALT levels caused by an ethanol diet. In addition, PT extract also prevented liver tissue damage caused by an ethanol diet. Thus, the effect of PT on ALD was found to be related to the inhibition of mitogen-activated protein kinase (MAP kinases) phosphorylation and TNF-α production.

## 1. Introduction

Microalgae are generally known to contain valuable nutrients such as encompassing fatty acids, sterols, terpenes, phenolic compounds, alkaloids, toxins, enzymes, polysaccharides, and pigments (lutein, +β-carotene) [1]. *Phaeodactylum tricornutum* (PT), a type of microalgae categorized as a diatom, is noted for its significant levels of eicosapentaenoic acid (EPA), which can account for up to 35% of its total fatty acid composition [2]. Furthermore, PT has garnered attention as a promising source of fucoxanthin, with its content potentially reaching levels up to one hundred times greater than that found in brown seaweeds [3,4]. Due to its unique carotenoid structure, fucoxanthin plays two key biological roles: scavenging free radicals and quenching singlet oxygen [5]. Fucoxanthin has been reported to prevent alcoholic liver disease (ALD) by reducing oxidative stress and inflammation [6].

The liver plays a central role in alcohol metabolism; however, alcohol can harm liver cells, potentially leading to ALD [7]. ALD is characterized by liver damage resulting from heavy drinking over a brief period [8,9]. This condition has become a significant public health concern globally, with its prevalence and associated morbidity rising annually due to increasing rates of alcohol abuse, which adversely affects physical health [10,11]. To address the rise in ALD, utilizing the properties of natural active substances instead of traditional drugs is considered a more effective and safer alternative.

This study aimed to assess the therapeutic effects of PT on alcoholic liver disease. To achieve this goal, we employed a model of liver injury induced by an alcohol-rich diet. This study verified the therapeutic effect of PT on alcoholic hepatitis, and it was shown that this effect was due to suppressing the production of TNF-α in the liver.

## 2. Results

### 2.1. Therapeutic Effect of PT on Alcoholic Hepatitis Model Mice

**Figure 1:**
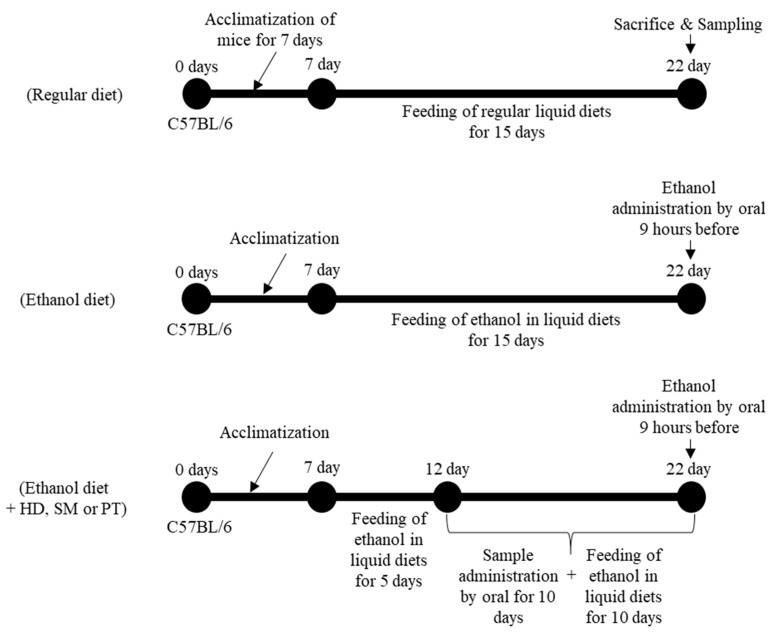
Outline of the experimental design for in vivo study the effect of PT extract on alcoholic liver disease.

**Figure 2:**
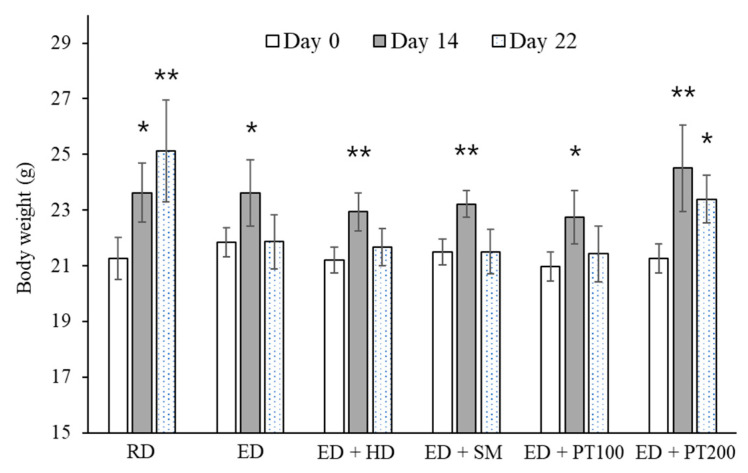
Effect of PT on weight loss in alcoholic hepatitis model mice. SM, HD, and PT were administered orally for 10 days to mice consuming an ED. Changes in the body weight of experimental animals during the experimental period were shown. The values represent the means ± SD. * *p* < 0.05 and ** *p* < 0.01 vs. Day 0.

**Figure 3:**
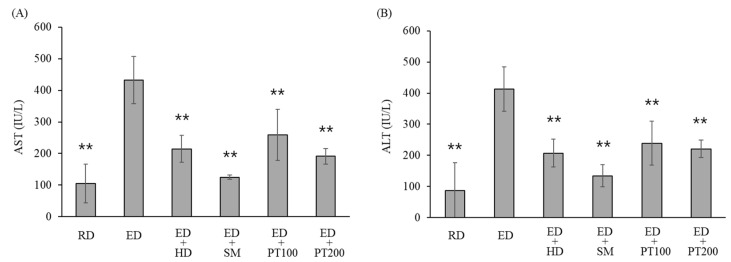
Effects of PT on the levels of serum ALT and AST in alcoholic hepatitis model mice. The ED fed mice were treated with HD, SM, or PT as described in Materials and Methods. The levels of serum AST (**A**) and AST (**B**) were determined by commercial reagent kits. The values represent the means ± SD and are expressed as IU/L of sera. ** *p* < 0.01 vs. ED group.

To explore the therapeutic effects of PT extract on alcoholic hepatitis, the model for ALD was induced by ad libitum feeding with the liquid diet containing ethanol for 15 days (Figure 1). The severity of alcohol-induced liver damage was assessed by body weight (Figure 2) and measuring serum liver enzyme (AST and ALT) levels (Figure 3). As positive controls to evaluate the activity of PT, *Hovenia dulcis* Thunb extract (HD) and *Silybum marianum* seed extract (SM), which are known to have preventive effects on alcoholic liver disease, were used.

Figure 2 shows the changes in body weight resulting from ethanol consumption. Mice on a regular diet (RD) exhibited a continuous increase in body weight throughout the experimental period, while those fed an ethanol diet (ED) showed a cessation of weight gain. On the 22nd day of the experiment, the body weight of mice on an ethanol diet was nearly the same as it was at the beginning of the experiment. In contrast, the body weight of mice orally administered PT (200 µg/kg) during the ethanol diet period increased by 2.13 g compared to their initial weight. As shown in Figure 2, the body weight of the mice on an ethanol diet decreased on the 22nd day of the experiment compared to the 14th day. However, the decrease in body weight for the mice on the ethanol diet with PT administration was less pronounced.

The levels of AST and ALT, which are indicators of liver function, were significantly increased by ethanol consumption (Figure 3). However, the levels of AST and ALT were reduced by nearly half with the PT extract, depending on the administration concentration (100 or 200 mg/kg). Among HD and SM, which were used as positive controls, the effect of SM was greater than that of HD.

### 2.2. Effects of Alcohol and PT Extract on Liver Histopathology

**Figure 4:**
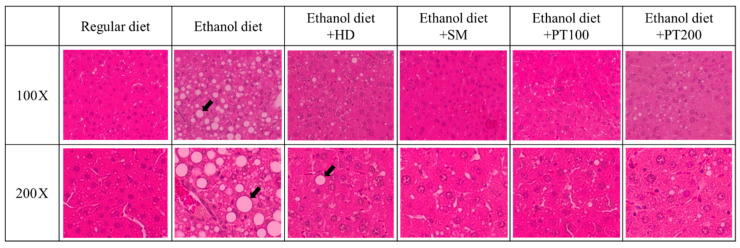
Liver histopathology of all experimental groups. These are images of liver histopathology using H&E straining at 100× and 200× magnification. The black arrows point out the areas of hepatocyte ballooning.

The therapeutic effects of PT on ALD were evaluated through histopathological analysis. The histopathological analysis of the liver was conducted following H&E staining. In mice fed ethanol, liver histopathology revealed notable hepatocyte ballooning (Figure 4). Following treatment with positive control extracts or PT, both groups showed significant reductions in the severity of hepatocyte ballooning compared to the ED group. The PT extract exhibited therapeutic effects similar to those of the SM extracts. In summary, PT extract helped prevent morphological changes in hepatocytes, revealing its potential to mitigate the pathological issues in liver tissue caused by alcohol consumption.

### 2.3. Effects of PT Extract on Production of Cytokines in Alcoholic Hepatitis Model Mice

**Figure 5:**
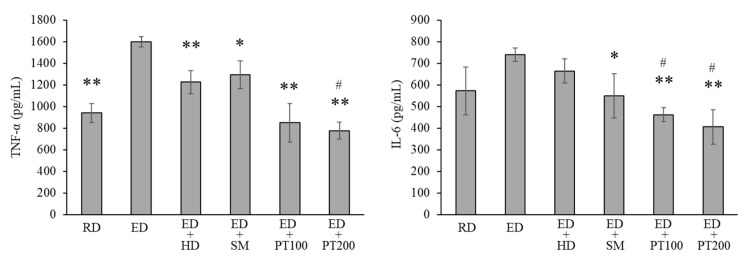
Effect of PT on the cytokine production in the alcoholic hepatitis model mice. Liver tissue extracts from animal models were utilized in ELISA assays to measure TNF-α and IL-6 levels. The data from three independent experiments, each of which was performed in triplicate. The values represent the means ± SD. * *p* < 0.05 and ** *p* < 0.01 vs. ED group. # *p* < 0.05 vs. ED + HD group.

To confirm the effect of PT on the inflammatory cytokines that increased in liver tissue due to alcohol consumption, the concentrations of TNF-α and IL-6 in liver tissue were measured using ELISA. Figure 5 illustrates that the levels of TNF-α and IL-6 were elevated due to the ED; however, these levels were significantly reduced following treatment with PT extracts (100 or 200 mg/kg). The elevated production of TNF-α resulting from alcohol consumption was also decreased by the positive controls, HD and SM, although their effect was less pronounced than that of PT. In contrast, the production of IL-6 was only reduced by PT and not by the treatments with HD and SM. The levels of TNF-α and IL-6 in serum were also measured, but the levels of these cytokines were below the measurable levels of the ELISA method in all experimental groups. Therefore, the levels of TNF-α and IL-6 in serum were not represented as results.

### 2.4. Therapeutic Effect of PT Extract on Severe Alcoholic Liver Injury

**Figure 6:**
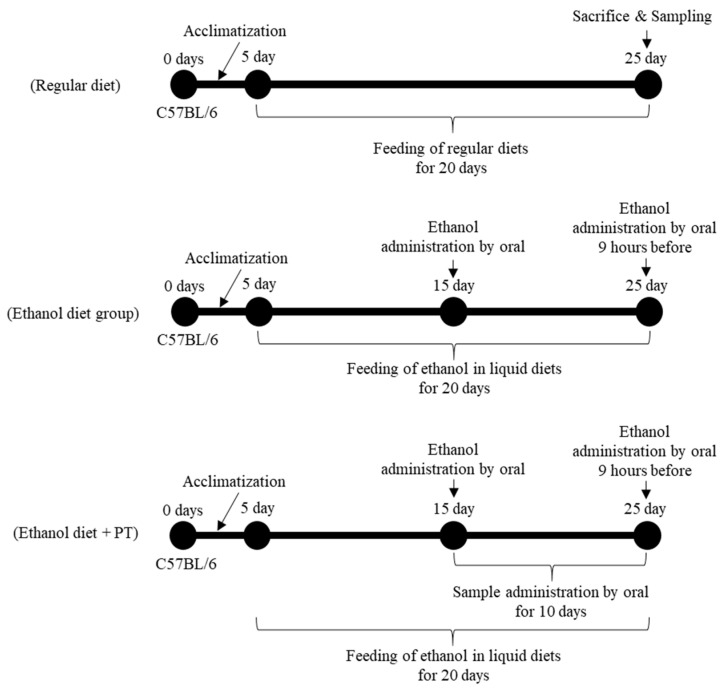
Outline of the experimental design for in vivo study the effect of PT extract on severe alcoholic liver disease.

**Figure 7:**
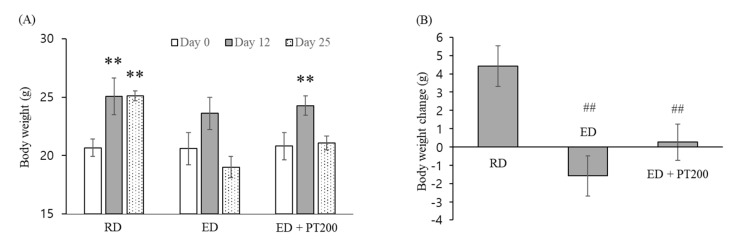
Effect of PT on weight loss in severe alcoholic hepatitis model mice. PT was administered orally for 10 days to mice consuming an ED. (**A**) Changes in the body weight of experimental animals during the experimental period. (**B**) Increased body weight on day 25 of the experiment. The values represent the means ± SD. ** *p* < 0.01 vs. Day 0. ## *p* < 0.01 vs. RD.

To confirm the PT effect on severe alcoholic hepatitis, the ED was administered for 5 days longer than in the previous experiment, and a 31.5% ethanol solution was orally administered one additional time compared to the previous experiment (Figure 6). As shown in Figure 7, mice fed an RD exhibited weight gain similar to that observed in the previous experiment (Figure 2). On the 25th day of the experiment, the body weight of mice on an ED decreased compared to the start of the experiment, and it was confirmed that the weight loss inhibition effect from PT administration was reduced compared to the previous experiment (Figure 2).

**Figure 8:**
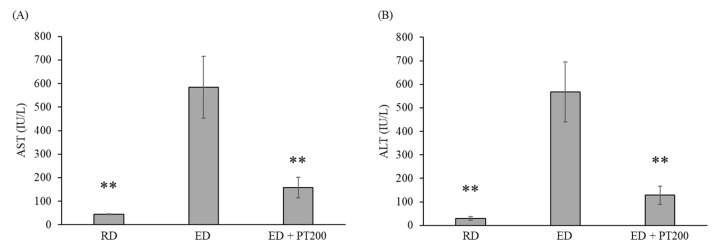
Effects of PT on serum ALT and AST levels in alcoholic hepatitis model mice. The levels of serum AST (**A**) and ALT (**B**) were determined by commercial reagent kits. The values represent the means ± SD and are expressed as IU/L of sera. ** *p* < 0.01 vs. ED group.

The AST and ALT levels of mice with severe ALD were approximately 600 IU/L (Figure 8). When PT extract was administered to these mice, the AST and ALT levels decreased by approximately 75%.

**Figure 9:**
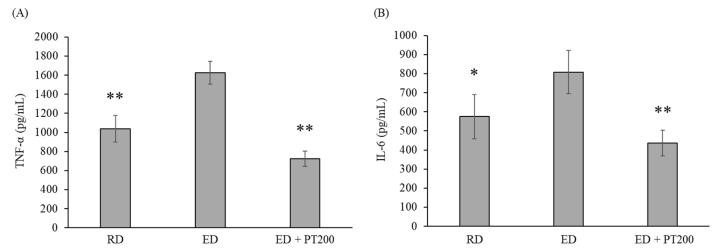
Effect of PT on the production of TNF-α (**A**) and IL-6 (**B**) in the alcoholic hepatitis model mice. The data from three independent experiments, each of which was performed in triplicate. The values represent the means ± SD. * *p* < 0.05 and ** *p* < 0.01 vs. ED group.

As shown in Figure 9, an ethanol diet increases TNF-α and IL-6 in liver tissue extracts, similar to the observations in Figure 5. These levels were significantly decreased following the administration of PT extracts (200 mg/kg).

### 2.5. Hepatoprotective Effect of PT Related to the MAP Kinase Pathway

**Figure 10:**
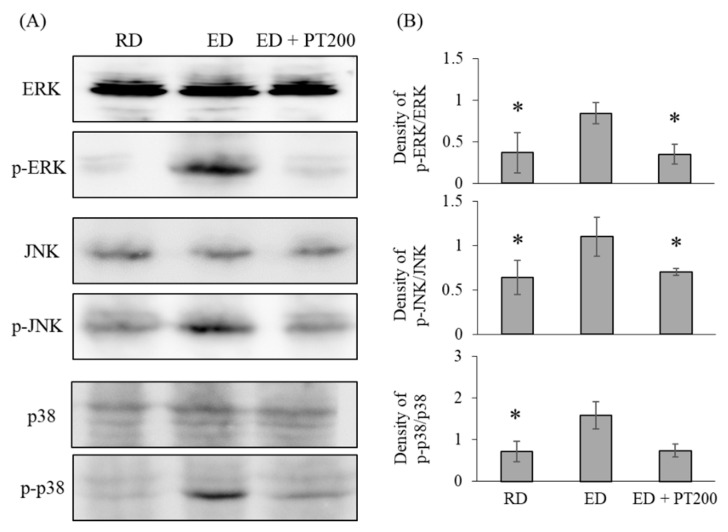
Effects of MAP kinases pathways on the hepatoprotective activity of PT. (**A**) The amount of phosphorylated ERK, JNK, and p38 in liver tissue extracts were analyzed by western blot analyses. (**B**) Quantification of band intensity of western blot. The data from three independent experiments, each of which was performed in triplicate. The values represent the means ± SD. * *p* < 0.05 vs. ED group.

Mitogen-activated protein kinases (MAP kinases) are known to be critical effectors in both physiological and pathophysiological hepatic inflammation. Therefore, this study investigated the influence of MAP kinase molecules (ERK, JNK, and p38) on the therapeutic efficacy of PT in the treatment of alcoholic liver disease. In liver tissue extracts from mice fed an ethanol diet (ED), the phosphorylation levels of ERK, JNK, and p38 were higher than those in rats fed a regular diet (RD), and these phosphorylation levels were reduced by PT treatment (Figure 10).

## 3. Discussion

The objective of this study was to illustrate the effects of PT extract on ALD. PT is a potentially valuable dietary supplement because PT-derived EPA and carotenoids are anti-inflammatory and antioxidant compounds [2,12]. EPA is an omega-3 fatty acid that may help with liver disease, including alcoholic hepatitis, and its effects are known to be through improving lipid metabolism and reducing inflammation [13,14]. Fucoxanthin, the primary carotenoid extracted from PT, has been shown to possess a variety of pharmacological effects [4,12]. Fucoxanthin is known for its powerful antioxidative properties and additional biological functions, including its anti-obesity, anti-inflammatory, and anti-cancer effects [15,16,17]. Moreover, fucoxanthin has been reported to enhance glycolipid metabolism in mice suffering from type 2 diabetes and to support ventricular rhythm and muscle performance in aging mouse models [18]. Zheng et al. found that fucoxanthin lessens oxidative stress from alcohol consumption by lowering oxidative product levels and promoting Nrf2-regulated antioxidant mechanisms [6]. Fucoxanthin also mitigates liver inflammation by blocking the signaling pathways activated by Toll-like receptor 4 [6]. The findings of this research indicate that PT, rich in EPA and fucoxanthin, holds significant potential for the creation of health foods aimed at preventing alcoholic liver damage.

Alcohol-related liver disease (ALD) represents a significant global contributor to chronic liver disorders, comprising a spectrum of conditions including uncomplicated steatosis, steatohepatitis, cirrhosis, and hepatocellular carcinoma. Although the last two decades have seen remarkable developments in ALD research, the precise pathophysiology of the disease continues to be unclear. Furthermore, although there are effective agents for ALD treatment, few have received pharmaceutical registration [19]. In this study, we used *Hovenia dulcis* Thunb extract (HD) and *Silybum marianum* seed extract (SM) as positive control substances.

In the results of this study (Figure 3), SM (milk thistle) was more effective than PT in lowering the AST and ALT levels elevated by alcohol consumption. SM is a herb and its derivatives have been used for centuries for the treatment of liver disease [20]. SM includes silymarin (composed primarily of silibin, silidianin, and silichristin), alkaloids, essential oils, histamine, lipids, saponins, sugars, mucilages, organic acid, tyramine, vitamins C, E, and K, and other flavonoids (quercitin, taxifolin, and dihydrokaempferol) [21,22]. Additionally, SM contains betaine and essential fatty acids, both of which have demonstrated anti-inflammatory properties [23]. It can be seen that the ingredients in SM are very similar to those in PT. The effect of PT on alcoholic hepatitis is likely due to its components being similar to those in SM.

In this study, MAP kinase pathways were suggested as a mechanism of the therapeutic activity of PT on alcoholic liver disease. MAP kinase cascades can be classified into at least three distinct types, which include the p42/44 MAPK, known as the extracellular signal-regulated kinases 1 and 2 (ERK1/2), the p38 MAP kinases, and the c-jun N-terminal kinase (JNK). The MAP kinase signaling cascade is crucial for initiating various cellular processes, including proliferation, differentiation, development, apoptosis, and responses to stress and inflammation. Recent evidence underscores the significant role of the MAP kinases family in mediating the effects of ethanol [24]. The way ethanol modulates the MAP kinases signaling pathway varies based on factors such as cell type, duration of exposure (acute or chronic), whether the cells are normal or transformed, and the specific agonist that activates the MAPK [24]. Among different cell types, the p42/44 MAPK pathway in hepatocytes is activated following ethanol treatment, with activation observed at both 1 h [25] and 24 h [26]. In contrast to p42/44 MAPK, the activation of JNK in response to ethanol is both strong and sustained [25]. In our results, the phosphorylation of MAP kinases (the activated form of MAP kinases) increased in the liver tissue of mice that consumed alcohol. However, PT had the effect of preventing the phosphorylation of MAP kinases induced by alcohol. In the liver tissue of mice fed alcohol, the ballooning phenomenon was confirmed to have increased compared to that of normal rats, but the phenomenon was decreased by the oral administration of PT. Based on these results, PT was found to inhibit liver tissue damage caused by alcohol. Excessive alcohol intake is known to induce tissue damage due to apoptosis of liver cell [27], and PT is expected to prevent this damage.

In addition, this study showed that alcohol consumption increased the production of cytokines (TNF-α and IL-6), which are indicators of inflammation, in hepatocytes, and that PT inhibited the production of cytokines increased by alcohol. IL-6 was reported to be elevated in the serum of inflammatory diseases patients such as rheumatoid arthritis, Castleman’s disease, and Crohn’s disease [28,29]. These findings contributed to the understanding that IL-6, like TNF-α and IL-1, serves as a marker of ongoing inflammation. However, the elevated levels of IL-6 are observed in some but not all patients with rheumatoid arthritis, and there was not always a direct correlation with the increased levels of TNF-α, IL-1, or other proinflammatory cytokines.

In this study, elevated levels of TNF-α were observed in the liver tissue of mice fed an ethanol diet. Alcoholic liver injury results from the direct effects of ethanol on hepatocytes, along with its impact on Kupffer cells and stellate cells [30,31]. Kupffer cells are bone marrow-derived cells that function like macrophages among white blood cells and are responsible for producing TNF-α in the liver. Elevated TNF-α production from activated Kupffer cells contributes to the cytokine imbalance observed in alcoholic liver injury [32,33,34]. Treatment of Kupffer cells with acetaldehyde (a metabolite of ethanol) led to an increased production of TNF, which was inhibited by blocking the p38 MAPK and p42/44 MAP kinases signaling pathways [35]. In addition, Kupffer cells from ethanol-fed mice produced more TNF than Kupffer cells from normal mice, and this TNF production was reduced by the p42/44 MAPK inhibitor [36,37]. These results suggest that the p38 MAPK and p42/44 MAPK pathways are involved in ethanol- and/or acetaldehyde-induced Kupffer cell activation and cytokine-mediated hepatocyte dysfunction in alcoholic liver injury, which is partially attributable to increased TNF-α production, supporting the idea that reductions in MAP kinase activity and TNF-α production can lead to decreased liver injury.

## 4. Materials and Methods

### 4.1. Materials and Reagents

*Hovenia dulcis* Thunb extract (HD) was obtained from Nutra Green Biotechnology Co., Ltd. (Shanghai, China) and *Silybum marianum* seed extract (SM) was obtained from Naturex (Avignon, France). The fucoxanthin standard was acquired from Sigma-Aldrich (St. Louis, MO, USA). Unless indicated, all other chemicals were also purchased from Sigma-Aldrich (St. Louis, MO, USA).

### 4.2. Cultivation of PT

In this study, PT (UTEX-646) was obtained from the Algal Culture Collection at the University of Texas at Austin (UTEX). PT was cultivated in seawater with the addition of F/2+Si medium. The F/2+Si medium contained the following components: 75 mg of NaNO_3_, 5.65 mg of NaH_2_PO_4_, 30 mg of Na_2_SiO_3_, 4.16 mg of Na_2_EDTA, 3.15 mg of FeCl_3_, 0.01 mg of CuSO_4_, 0.022 mg of ZnSO_4_, 0.01 mg of CoCl_2_, 0.18 mg of MnCl_2_, 0.006 mg of Na_2_MoO_4_, 0.0005 mg of vitamin B_12_, 0.1 mg of vitamin B_1_, and 0.0005 mg of biotin.

Before use, all media were sterilized at 121 °C for 15 min. Seawater was disinfected with sodium hypochlorite (NaOCl) in a 50-ton photobioreactor (PBR), followed by the addition of the F/2+Si stock solution. PT cultures were then inoculated and incubated for approximately seven days. After incubation, PT biomass was harvested using continuous centrifugation.

### 4.3. Extraction Procedure of PT Extract

Wet PT biomass was extracted by continuous stirring for 15 h in the presence of 95% ethanol, adjusting the final ethanol concentration to 70%. The resulting mixture was subjected to vacuum-assisted filtration using filter paper (Whatman, pore size 5 μm, Bucks, UK). The filtrate was then homogenized (13,000 rpm, 5 min) with excipients (β-cyclodextrin; ES Food Ingredients, Gunpo, Republic of Korea). The homogenized solution was concentrated under reduced pressure (40 °C, 100 mbar, 100 rpm) using a vacuum rotary evaporator (Rotavapor R-300, BUCHI, Flawil, Switzerland). The concentrated extract was then thermally sterilized at 70 °C for 30 min and subsequently lyophilized to obtain the final product.

### 4.4. Characterization of PT Extract

To evaluate the quality and uniformity of the PT extract, analyses were conducted to determine fucoxanthin concentration, the presence of *Escherichia coli*, and the levels of heavy metals, including lead, arsenic, cadmium, and mercury. These tests were carried out at the Korea Health Supplement Institute (Seoul, Republic of Korea), an accredited laboratory designated by the Ministry of Food and Drug Safety (MFDS, Osong, Republic of Korea). The testing process adhered to the official analytical methods outlined in the Korean Food Standards and Specifications [38].

**Figure 11:**
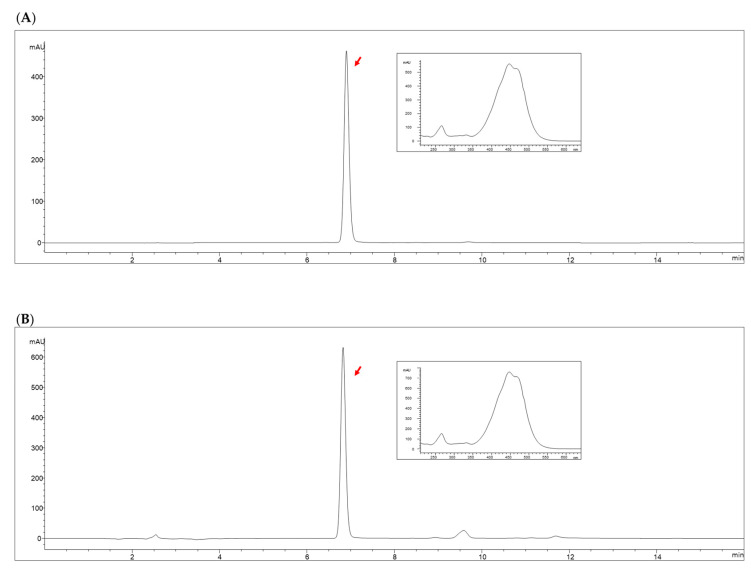
HPLC chromatograms illustrating the fucoxanthin standard (**A**) and PT extract (**B**) measured at 450 nm. The inset shows the absorption spectrum corresponding to the fucoxanthin peak. Red arrows indicate the fucoxanthin peak in each chromatogram.

The analysis results from three independent PT extract batches showed fucoxanthin concentrations of 18.14, 17.31, and 18.33 mg/g (Figure 11). The detected levels of heavy metals were as follows: lead (Pb) at 0.0313, 0.0312, and 0.0323 ppm; arsenic (As) at 0.0124, 0.0152, and 0.0168 ppm; cadmium (Cd) at 0.0344, 0.0368, and 0.0375 ppm; and mercury (Hg) at 0.0047, 0.0050, and 0.0047 ppm.

For quality assurance, the PT extract must meet the following regulatory thresholds: Fucoxanthin concentration should range between 14.33 and 21.50 mg/g, while heavy metal limits must remain below the specified values—1.0 ppm for lead (Pb), 1.0 ppm for arsenic (As), 0.3 ppm for cadmium (Cd), and 0.5 ppm for mercury (Hg). Additionally, *Escherichia coli* must be entirely absent in all tested samples, and the total pheophorbide content should not exceed 1000 ppm.

### 4.5. Analysis of Fucoxanthin

Fucoxanthin content was quantified using high-performance liquid chromatography (HPLC) with an Agilent 1260 system (Agilent Technologies, Santa Clara, CA, USA), following a previously validated method [39]. Chromatographic separation was performed on a CAPCELL PAK C18 MG II column (5 μm particle size, 250 × 4.6 mm I.D.; Phenomenex, CA, USA). The mobile phase consisted of acetonitrile (solvent A) and water (solvent B), with a flow rate of 1.0 mL/min. The gradient elution profile was programmed as follows: initial composition of 90:10 (A/B), increased to 100:0 over 8 min, maintained at 100:0 for 3 min, and then adjusted to 80:20 over the subsequent 5 min. Detection was carried out at 450 nm using a UV-Vis detector (1260 DAD HS, Agilent Technologies, Santa Clara, CA, USA). Quantification was achieved by integrating peak areas and comparing them to a calibration curve prepared with fucoxanthin standard solutions at concentrations of 1, 5, 10, 50, 100, and 200 μg/mL.

### 4.6. Animals

Male C57BL/6J mice, aged seven weeks and weighing 21.0 ± 3.0 g, were acquired from Dooyeol Biotech in Seoul, Korea. The animals were kept in standard laboratory settings at 23 ± 2 °C, underwent a 12 h light-dark cycle, and had uninterrupted access to food and water. All experiments were carried out in accordance with the established guidelines of the Committee for the Purpose of Control and Supervision of Experiments on Animals and the National Institutes of Health regarding the proper use of experimental animals. The experimental protocol received approval from the Ethics Committee for Animal Experimentation of Chungbuk National University (Project identification code: CBNUA-2017-22-02; date of approval: 11 October 2022; Republic of Korea).

### 4.7. Ethanol Diet Induced Live Injury Model

Forty eight C57BL/6J mice were categorized into six groups, with eight mice in each group, as illustrated in Table 1.

As shown in Figure 1, an alcoholic liver model was established using an ethanol liquid diet (ED), as previously detailed [40]. Briefly, for the therapeutic activity of PT on alcoholic liver disease, C57BL/6J mice were fed ethanol diet for 5 days, and then administrated PT, SM, and HD orally for 10 days. A 31.5% ethanol solution was administered orally 9 h before autopsy. Before the autopsy, blood was collected from experimental mice, serum was separated, and the separated serum was used to measure aspartic transaminase (AST) and alanine transaminase (ALT). Liver tissues were obtained from each animal at euthanasia. The right lateral lobe of the liver was sliced into small fragments using scalpel blades, and approximately 0.2 g of the tissue was rapidly frozen in liquid nitrogen and kept at −80 °C until further analysis for cytokines and western blotting.

To confirm the therapeutic effect and mechanism of PT for severe alcoholic hepatitis, C57BL/6J mice were fed an ethanol liquid diet (ED) for 20 days (Figure 6). A 31.5% ethanol solution was administered orally on day 15 of the experimental period and 9 h before the autopsy. Regular liquid diet (RD) was prepared as described in Table 1 and fed for 20 days. PT extract (200 mg/kg) was administered orally for 10 days before the autopsy.

### 4.8. Determination of Serum ALT and AST

Blood samples were collected, and serum was obtained through centrifugation at 4 °C for 10 min at 3000 rpm. Serum levels of ALT and AST were measured following the guidelines outlined in the reagent kits (ALT: ab105134; AST: ab105135, Abcam, Cambridge, UK). The results were reported in IU/L of serum.

### 4.9. Cytokine Assay

Liver tissue was thawed on ice, and approximately 0.2 mL of glass beads (Sigma-Aldrich), along with 1 mL of phosphate-buffered saline (PBS) containing protease inhibitors (Sigma-Aldrich), were added to prevent degradation during thawing. The liver samples were then homogenized three times for 5 min at 4 °C using a Bullet-Blender (Next Advance, Averill Park, NY, USA). Following this, the homogenates underwent two sequential centrifugation steps (164× *g*, 4 °C, for 10 min). The supernatants, stored at −80 °C, were analyzed for cytokine levels using electrochemiluminescence within one week of processing the tissue. The concentrations of TNF-α and IL-6 (TNF-α: SMTA00B; IL-6: SM6000B, R&D Systems, Minneapolis, MN, USA) in the serum were measured using enzyme-linked immunosorbent assays (ELISA), following the manufacturer’s protocols.

### 4.10. Western Blot Analysis

Western blot samples were prepared in a manner similar to that used for cytokine assays and were resolved using 12% SDS-PAGE. The samples were then transferred to a PVDF membrane (Immobilon-P, Millipore, Billerica, MA, USA). The membranes were blocked with Tris-buffered saline (10 mM Tris-Cl, pH 7.4) containing 0.5% Tween 20 and 5% non-fat dry milk for 1 h, followed by incubation with the primary antibody, diluted at a ratio of 1:100 in the blocking solution for 5 h. Afterward, the membranes were treated with a horseradish peroxidase (HRP)-conjugated secondary antibody diluted at a ratio of 1:1000 for 1 h. Primary antibodies (p38 #9212, p-p38 #9211, ERK #4695, p-ERK #4370, JNK #9252, and p-JNK #9251) and secondary antibodies (anti-rabbit IgG HRP-linked antibody, #7074) were purchased from Cell Signaling Technology (Boston, MA, USA). Protein bands were visualized using ECL (Amersham Pharmacia, Biotech, Piscataway, NJ, USA). The intensity of the protein bands was assessed and analyzed using Image Lab 6.1 software. (Bio-Rad, Hercules, CA, USA).

### 4.11. Histological Analysis

Formalin-fixed liver tissues were embedded in paraffin. Tissue sections of 4 μm thickness were stained with hematoxylin and eosin (H&E) solution (ab245880, Abcam). To prepare the tissue sections, first deparaffinize and hydrate them in distilled water. Then, apply an adequate amount of Hematoxylin (Mayer’s Modification) to completely cover the tissue section and incubate for 5 min, followed by rinsing the slide in two changes of distilled water. Next, apply an adequate amount of Eosin Y Solution (Modified Alcoholic) to completely cover the tissue section and incubate for 2–3 min. The stained liver samples were then observed under a light microscope (Olympus, Tokyo, Japan).

### 4.12. Statistical Analysis

IBM SPSS Statistics (version 27; SPSS Inc., Chicago, IL, USA) was used to analyze the data. In vivo experiments were conducted using eight mice per group, and data were presented as means ± standard deviation (SD). Differences between different groups were analyzed using a one-way ANOVA followed by Tukey’s multiple comparison post hoc test. *p* < 0.05 was considered statistically significant.

## 5. Conclusions

This study offers substantial evidence that PT has a positive impact on ALD. These results contribute important insights into the potential of PT as a therapeutic option for managing ALD. In this study, we found that the inhibition of MAP kinase signaling is involved in the inhibitory effect of PT on ALD. But the inhibition of MAP kinase signaling alone is limited in explaining the mechanisms of all the effects of PT presented in this paper. Therefore, in future studies, we plan to more clearly identify the mechanism of PT’s effect on ALD.

## Figures and Tables

**Table 1:** Comparison of the therapeutic effect of PT on alcoholic hepatitis with the positive control group.

Number	Group	Explanation
1	Regular liquid diet (RD)	To prepare the regular liquid diet, 222 g of the diet mix powder should be added to one liter of water. The mixture must be blended for approximately 30 s using a blender. It is essential that the liquid diet be prepared fresh on a daily basis.
2	Ethanol liquid diet (ED)	To prepare the ethanol liquid diet, 132 g of the diet mix powder should be added to 54 g of 95% ethanol (about 67 mL), and then water to 1 L. The mixture must be blended for approximately 30 s using a blender. Ethanol liquid diet is prepared just before feeding.
3	ED + SM	*Silybum marianum* seed extract (150 mg/kg) is administered orally during the period of ED consumption.
4	EP + HD	*Hovenia dulcis* Thunb extract (200 mg/kg) is administered orally during the period of ED consumption.
5	EP + PT100	PT extract (100 mg/kg) is administered orally during the period of ED consumption.
6	EP + PT200	PT extract (200 mg/kg) is administered orally during the period of ED consumption.

## Data Availability

The corresponding authors can make any materials available upon request. The aggregate data from the referenced datasets are available from the corresponding authors on reasonable request.
